# An updated meta-analysis of 23 case-control studies on the association between miR-34b/c polymorphism and cancer risk

**DOI:** 10.18632/oncotarget.16322

**Published:** 2017-03-17

**Authors:** Hua Li, Shuling Diao, Jingsen Li, Baoxin Ma, Shuanghu Yuan

**Affiliations:** ^1^ Department of Oncology, The Affiliated Hospital of Binzhou Medical University, Binzhou, Shandong 256603, China; ^2^ Department of Cardiology, The Affiliated Hospital of Binzhou Medical University, Binzhou, Shandong 256603, China; ^3^ Department of Radiotherapy, Shandong Cancer Hospital and Institute, Jinan, Shandong 250117, China

**Keywords:** rs4938723, polymorphism, cancer risk, systematic review, meta-analysis

## Abstract

The association between in microRNA-34b/c gene rs4938723 polymorphisms and cancer risk remains inconclusive. This meta-analysis was performed to analyze the association between microRNA-34b/c rs4938723 polymorphism and risk for cancer development. In total, 304 studies from PubMed, Embase, Web of Science, Wanfang, and Chinese National Knowledge Infrastructure databases were examined, and 23 studies were included in this meta-analysis. The 23 selected studies involved 10,812 cancer cases and 11,719 controls. Odds ratios (ORs) and 95% confidence intervals (CIs) were calculated to measure the strength of the association. Our results indicate a significant association between the rs4938723 polymorphism and cancer risk in the overdominant model (P heterogeneity = 0.018, OR = 1.093, and 95% CI = 1.015–1.177 for CT vs. CC/TT). Using a stratified subgroup analysis, rs4938723 polymorphisms were associated with an increased risk for hepatocellular carcinoma, but decreased risk for colorectal, gastric, and esophageal squamous cell cancer. These findings indicate that the rs4938723 gene is a susceptible locus for cancer.

## INTRODUCTION

Cancer is one of the most serious public health problems worldwide [1]. Intensive efforts have been undertaken to improve the efficacy of cancer diagnosis and therapy; however, the overall survival time of cancer patients is still short [2, 3]. Further studies on the risk factors, biomarkers, and therapeutic targets for cancer should reduce the cancer burden. Emerging evidence has revealed that genetic factors, such as single nucleotide polymorphisms (SNPs), influence cancer development, treatment efficacy, and survival time of cancer patients [2].

Mature microRNAs (miRNAs) are a class of endogenous, single-stranded, and non-protein-coding small RNAs, which play an important role in tumorigenesis and cancer progression [4–6]. The SNPs in the genomic miRNA sequences influence miRNA-dependent regulation and alter tumor susceptibility [7, 8].

The microRNA-34 (miR-34) family comprises three miRNAs, namely, miR-34a, miR-34b, and miR-34c [9]. The miR-34b/c gene rs4938723 has been associated with hepatocellular and colorectal cancer [10–12]. However, updated, recent meta-analyses of the rs4938723 association with cancer risk have been limited. In this study, we have systematically reviewed the published data, and integrated all published studies to evaluate the association between rs4938723 polymorphism and cancer risk.

## RESULTS

### Study characteristics

**Figure 1:**
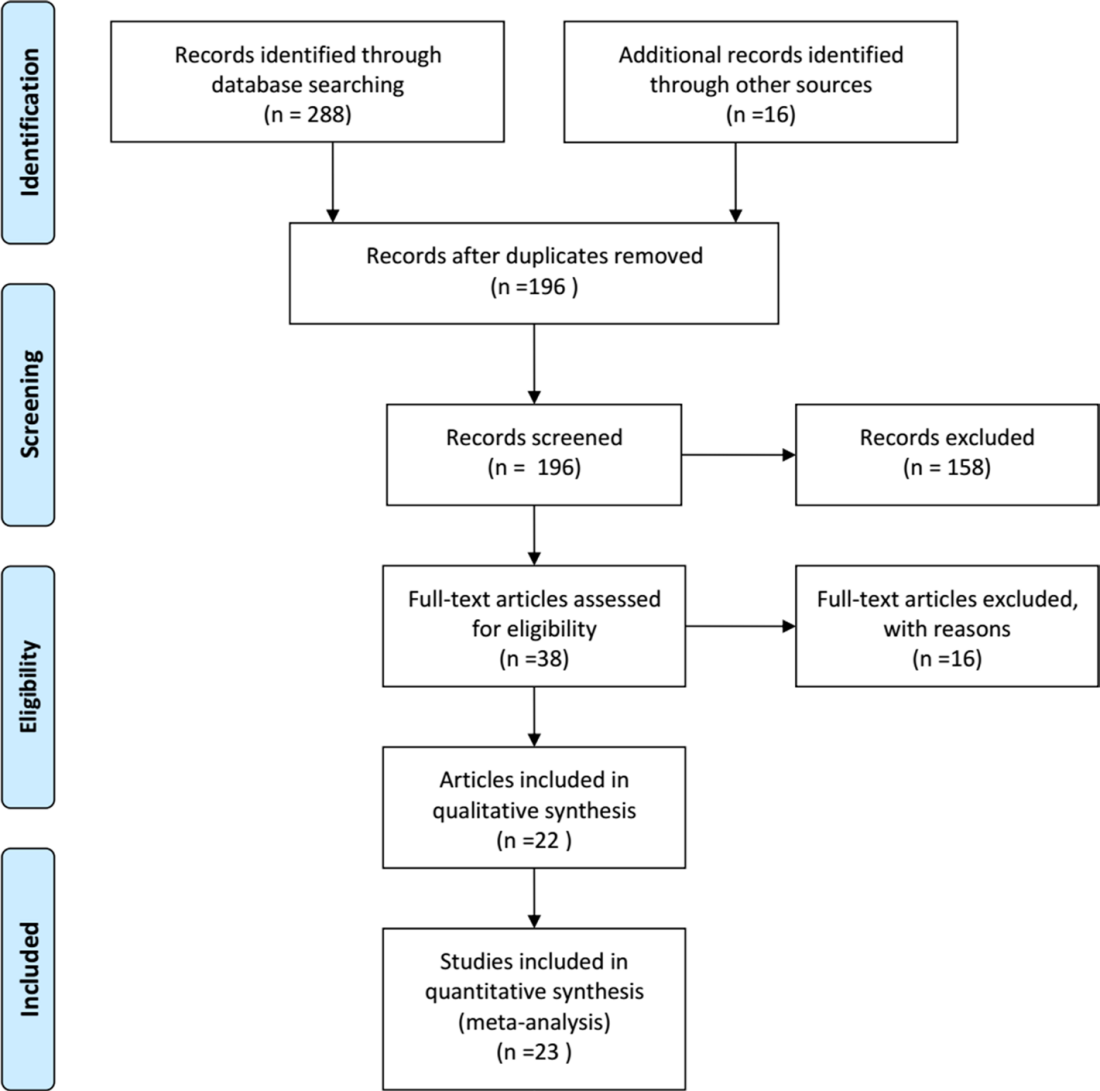
Flow chart of studies selection in this meta-analysis

Relevant studies from the PubMed, Embase, Web of Science, Wanfang, and Chinese National Knowledge Infrastructure databases were examined. A flowchart of the selected studies is presented in Figure 1. In total, 304 studies were searched (288 from the databases and 16 using a manual search). 108 duplicated retrieval articles were excluded from this study. In addition, 158 records were excluded because of improper titles and/or abstracts. 38 eligible studies were selected for a detailed evaluation. From these, 16 articles were excluded from this study according to the inclusion and exclusion criteria. Finally, 22 original articles containing 23 studies were included in the meta-analysis [7, 13–33]. The characteristics of the studies included are listed in Table 1. From the 23 studies, 20 were on patients of Asian descent, two were on patients of Caucasian descent, and one was on patients of African descent.

**Table 1:** Main characteristics of studies included in the meta-analysis

Author	Year	Country	Ethnicity	Cancer type	Genotyping methods	Cases	Controls	Case					Control					P value for HWE test in our controls	Quality
								TT	CT	CC	T	C	TT	CT	CC	T	C		
Hashemi-1	2016	Iran	Asian	PC	PCR-RFLP	151	152	85	56	10	226	76	109	38	5	256	48	0.46	8
Hashemi-2	2016	Iran	Asian	LL	PCR-RFLP	110	120	77	31	2	185	35	62	52	6	176	64	0.24	5
Sanaei	2016	Iran	Asian	BC	PCR-RFLP	263	221	125	115	23	365	161	100	106	15	306	136	0.06	4
Yuan	2016	China	Asian	CC	PCR-RFLP	328	568	117	175	36	409	247	242	258	68	742	394	0.95	7.5
Zhu	2015	China	Asian	ESCC	MALDI-TOF MS	237	274	113	99	25	325	149	122	122	30	366	182	0.95	8
Chen	2015	China	Asian	PTC	PCR-RFLP	784	1006	271	402	111	944	624	456	451	99	1363	649	0.41	8
Pan	2015	China	Asian	GC	PCR-RFLP	197	289	102	76	19	280	114	121	137	31	379	199	0.4	7.5
Tong	2015	China	Asian	LL	Taqman assay	570	673	254	281	35	789	351	301	296	76	898	448	0.8	10
Yang	2014	China	Asian	GC	PCR-RFLP	419	402	193	186	40	572	266	156	184	62	496	308	0.53	7.5
Zhang-1	2014	China	Asian	RCC	Taqman assay	710	760	302	324	84	928	492	352	344	64	1048	472	0.12	8.5
Zhang-2	2014	China	Asian	ESCC	Taqman assay	1109	1275	489	536	84	1514	704	569	573	133	1711	839	0.52	10
OH	2014	Korea	Asian	CRC	PCR-RFLP	545	428	272	233	40	777	313	216	171	41	603	253	0.4	7
Li	2013	China	Asian	NPC	PCR-RFLP	217	360	82	104	31	268	166	168	155	37	491	229	0.89	9
Han	2013	China	Asian	HCC	Fluorescent-probe qRT-PCR	1013	999	451	444	118	1346	680	456	424	119	1336	662	0.18	10
Tian	2013	China	Asian	OS	Taqman assay	133	133	41	62	30	144	122	62	53	18	177	89	0.23	9
Gao	2013	China	Asian	CRC	PCR-RFLP	347	488	175	144	28	494	200	216	210	62	642	334	0.33	7.5
Bensen-1	2013	America	African	BC	Genotyping array	742	658	362	317	63	1041	443	343	257	58	943	373	0.32	8
Bensen-2	2013	America	Caucasian	BC	Genotyping array	1203	1088	496	563	144	1555	851	430	503	155	1363	813	0.69	8
Yin	2013	China	Asian	ESCC	PCR-LDR	629	686	277	278	45	832	368	310	290	73	910	436	0.68	9.5
Son	2013	Korea	Asian	HCC	PCR-RFLP	157	201	69	75	13	213	101	110	74	17	294	108	0.37	7
Xu	2011	China	Asian	HCC	PCR-RFLP	502	549	204	236	62	644	360	266	229	54	761	337	0.65	10
Krzysztof	2011	Poland	Caucasian	LL	PCR-RFLP	195	200	79	88	28	246	144	98	83	19	279	121	0.81	7
You	2011	China	Asian	ESCC	MALDI-TOF MS	251	189	120	103	28	343	159	88	86	15	262	116	0.34	7

NPC nasopharyngeal carcinoma, PC prostate cancer, HCC hepatocellular carcinoma, OS osteosarcoma, CRC colorectal cancer, BC breast cancer, ESCC esophageal squamous cell cancer, LL lymphocytic leukemia, RCC renal cell cancer, CC cervical cancer, GC gastric cancer, PTC papillary thyroid carcinoma,

PCR-RFLP polymerase chain reaction–restriction fragment length polymorphism, PCR-LDR polymerase chain reaction–ligation detection reaction, MALDI-TOF MS matrix-assisted laser desorption/ionization time-of-flight mass spectrometry.

The 23 selected studies involved 10,812 cases and 11,719 controls. Among these studies, four studies were related to esophageal squamous cell cancer, three to hepatocellular cancer, three to breast cancer, three to lymphocytic leukemia, two to colorectal cancer, and two studies were related to gastric cancer. Only one study each was related to cervical cancer, papillary thyroid carcinoma, renal cell cancer, nasopharyngeal carcinoma, prostate cancer, and osteosarcoma. The genotype distribution in the controls was compatible with the Hardy–Weinberg equilibrium (HWE) in all 23 studies. The quality scores for the individual studies ranged from 4 to 10, and the median score was 8.0.

### Main analysis results

**Figure 2:**
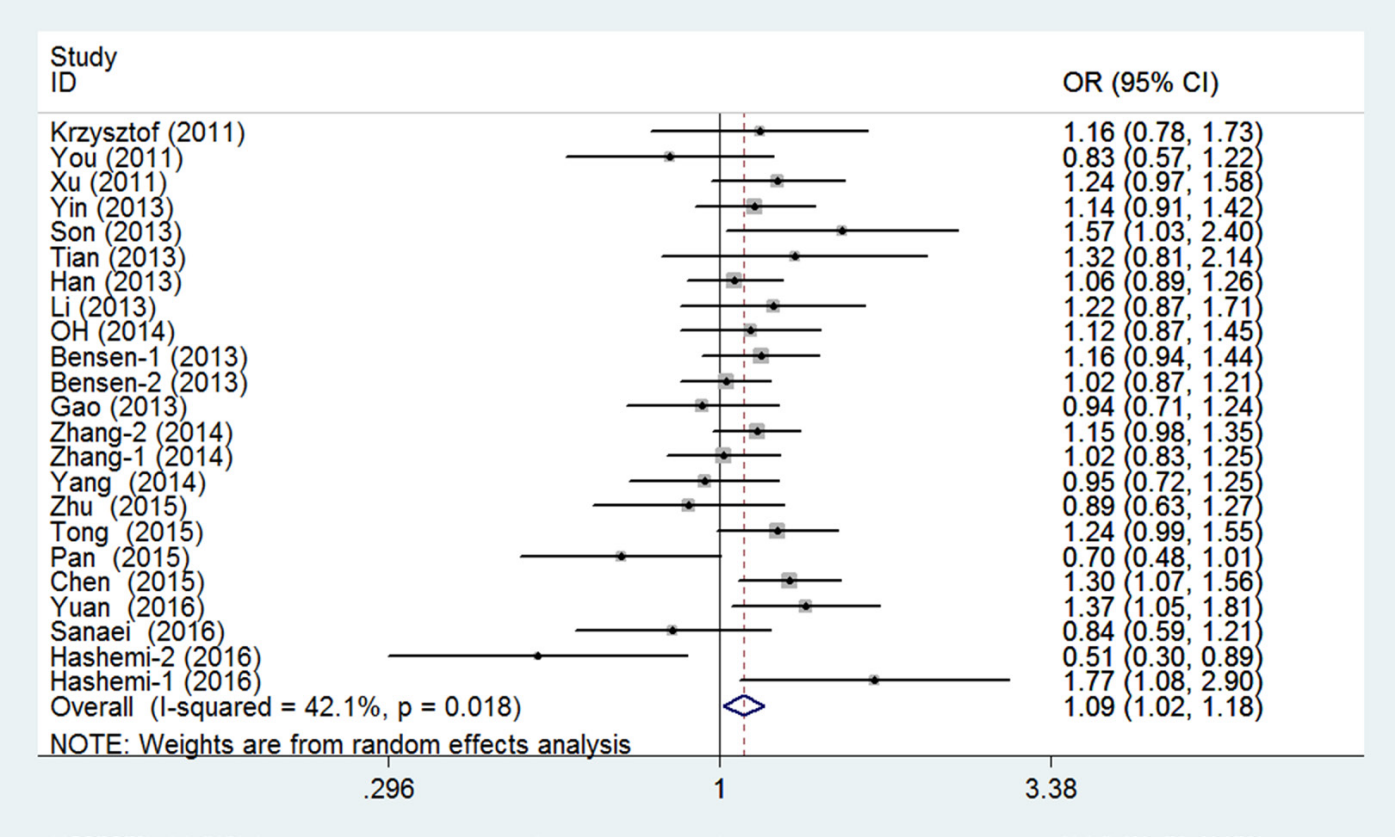
Forest plots of the association between miR-34b/c gene rs4938723 and cancer susceptibility (overdominant model)

In the meta-analysis of the 23 eligible studies, genotype CT was significantly associated with cancer susceptibility in the overall population (overdominant model CT versus CC/TT: P_H_ = 0.018, OR = 1.093, and 95% CI = 1.015–1.177), as shown in Table 2 and Figure 2. No association between rs4938723 polymorphism and cancer risk was observed in the allele, genotype, dominant, and recessive models.

**Table 2:** ORs and 95% CI for cancer risk and pri-miR-34b/c polymorphism (rs4938723 T > C) under different genetic models

Comparison	Subgroup	*N*	Heterogeneity test		*Z* test*P_Z_*	Publication bias		OR and 95%CI	
			P_H_	I^2^(%)		*P_B_*	*P_E_*	Fixed model	Random model
C vs. T	Overall	23	0	75.7	0.356	0.492	0.677	1.031 (0.991–1.072)	1.041(0.956–1.134)
	Asian	20	0	77.2	0.470			1.037 (0.993–1.084)	1.037(0.940–1.143)
	Caucasian	2	0.018	82.1	0.668			0.969 (0.867–1.084)	1.086 (0.746–1.580)
	LL	3	0.002	83.9	0.571			0.929 (0.808–1.068)	0.886 (0.582–1.349)
	ESCC	4	0.898	0	0.225			0.947 (0.867–1.034)	0.947 (0.867–1.034)
	HCC	3	0.113	54.2	0.036			1.114 (1.007–1.233)	1.151 (0.974–1.360)
	CRC	2	0.154	50.9	0.058			0.870 (0.754–1.005)	0.867 (0.706–1.066)
	BC	3	0.304	16.1	0.556			0.973 (0.888–1.066)	0.978 (0.881–1.085)
	GC	2	0.843	0	0.001			0.758 (0.643–0.893)	0.758 (0.643–0.893)
CT vs. TT	Overall	23	0	60.6	0.061	0.833	0.825	1.099 (1.040–1.162)	1.094 (0.996–1.203)
	Asian	20	0	64.1	0.118			1.107 (1.041–1.177)	1.091 (0.978–1.217)
	Caucasian	2	0.192	41.2	0.853			1.015 (0.863–1.195)	1.061 (0.809–1.392)
	LL	3	0.011	77.9	0.788			1.047 (0.865–1.267)	0.936 (0.578–1.515)
	ESCC	4	0.599	0	0.552			1.038 (0.919–1.172)	1.038 (0.919–1.172)
	HCC	3	0.121	52.6	0.016			1.191 (1.033–1.373)	1.250 (0.993–1.573)
	CRC	2	0.222	32.9	0.741			0.967 (0.795–1.177)	0.964 (0.758–1.226)
	BC	3	0.289	19.5	0.760			1.020 (0.897–1.161)	1.020 (0.878–1.185)
	GC	2	0.381	0	0.018			0.755 (0.598–0.953)	0.755 (0.598–0.953)
CC vs. TT	Overall	23	0	72.1	0.794	0.460	0.622	0.994 (0.908–1.088)	1.025 (0.851–1.234)
	Asian	20	0	73.9	0.872			1.008 (0.911–1.115)	1.018 (0.822–1.261)
	Caucasian	2	0.022	80.8	0.737			0.904 (0.710–1.151)	1.146 (0.517–2.539)
	LL	3	0.005	81.3	0.568			0.745 (0.529–1.048)	0.744 (0.270–2.051)
	ESCC	4	0.345	9.5	0.026			0.787 (0.638–0.972)	0.794 (0.632–0.998)
	HCC	3	0.285	20.4	0.221			1.150 (0.919–1.439)	1.172 (0.895–1.535)
	CRC	2	0.342	0	0.015			0.658 (0.470–0.923)	0.661 (0.471–0.928)
	BC	3	0.386	0	0.300			0.897 (0.730–1.102)	0.896 (0.729–1.102)
	GC	2	0.400	0	0.004			0.584 (0.405–0.842)	0.584 (0.405–0.842)
CC/CT vs. TT	Overall	23	0	70.9	0.145	0.792	0.890	1.078 (1.022–1.137)	1.081 (0.974–1.199)
	Asian	20	0	73.1	0.229			1.086 (1.024–1.152)	1.076 (0.955–1.213)
	Caucasian	2	0.060	71.8	0.638			0.992 (0.850–1.157)	1.100 (0.738–1.640)
	LL	3	0.005	81.5	0.688			0.986 (0.821–1.184)	0.902 (0.545–1.493)
	ESCC	4	0.891	0	0.881			0.991 (0.882–1.113)	0.991 (0.882–1.113)
	HCC	3	0.090	58.6	0.065			1.184 (1.034–1.354)	1.248 (0.987–1.577)
	CRC	2	0.157	50	0.288			0.904 (0.750–1.089)	0.899 (0.690–1.171)
	BC	3	0.287	19.9	0.958			0.997 (0.882–1.127)	0.999 (0.866–1.153)
	GC	2	0.664	0	0.003			0.715 (0.574–0.892)	0.715 (0.574–0.892)
CC vs. CT/TT	Overall	23	0	64.0	0.704	0.561	0.557	0.946 (0.868–1.031)	0.970 (0.830–1.134)
	Asian	20	0	66.7	0.702			0.954 (0.867–1.050)	0.965 (0.806–1.157)
	Caucasian	2	0.049	74.2	0.829			0.897 (0.716–1.124)	1.073 (0.564–2.043)
	LL	3	0.008	79.2	0.521			0.703 (0.507–0.976)	0.739 (0.294–1.860)
	ESCC	4	0.164	41.2	0.013			0.774 (0.633–0.947)	0.815 (0.612–1.086)
	HCC	3	0.493	0	0.579			1.062 (0.859–1.314)	1.062 (0.859–1.314)
	CRC	2	0.519	0	0.016			0.672 (0.485–0.930)	0.674 (0.486–0.934)
	BC	3	0.387	0	0.241			0.890 (0.733–1.081)	0.889 (0.732–1.081)
	GC	2	0.254	23.1	0.022			0.667 (0.471–0.943)	0.678 (0.452–1.017)
CT vs.CC/TT	Overall	23	0.018	42.1	0.018	0.428	0.423	1.101 (1.044–1.161)	1.093 (1.015–1.177)
	Asian	20	0.008	48.5	0.051			1.105 (1.042–1.172)	1.090 (1.000–1.189)
	Caucasian	2	0.57	0	0.597			1.042 (0.895–1.213)	1.042 (0.895–1.213)
	LL	3	0.014	76.5	0.849			1.103 (0.919–1.325)	0.957 (0.611–1.501)
	ESCC	4	0.303	17.6	0.190			1.081 (0.962–1.214)	1.067 (0.932–1.222)
	HCC	3	0.195	38.9	0.035			1.157 (1.010–1.324)	1.192 (0.986–1.441)
	CRC	2	0.357	0	0.729			1.034 (0.856–1.249)	1.034 (0.856–1.249)
	BC	3	0.299	17.1	0.495			1.044 (0.923–1.179)	1.041 (0.950–1.197)
	GC	2	0.193	41.1	0.140			0.847 (0.680–1.056)	0.833 (0.620–1.119)

*P_H_*: *P*-value of heterogeneity test; *P_Z_*: *P*-value of *Z* test; *P_B_*: *P*-value of Begg's test; *P_E_*: *P*-value of Egger's test; OR: odds ratio; 95% CI: 95% confidence interval; *N*: number of comparisons.

### Subgroup analysis results

In Asian and Caucasian populations, no significant association was observed in any genetic models. For hepatocellular carcinoma, the rs4938723 polymorphism was associated with an increased cancer risk in the comparison model (allele C versus T: P_H_ = 0.113, OR = 1.114, and 95% CI = 1.007–1.233), genotype model (CT versus TT: P_H_ = 0.121, OR = 1.191, and 95% CI = 1.033–1.373), and overdominant model (CT vs. CC/TT: P_H_ = 0.195, OR = 1.157, and 95% CI = 1.010–1.324). For lymphocytic leukemia, no association was observed in any genetic models. The rs4938723 polymorphism decreased the risk for colorectal cancer in the genotype (CC vs. TT: P_H_ = 0.342, OR = 0.658, and 95% CI = 0.470–0.923) and recessive models (CC vs. CT/TT: P_H_ = 0.519, OR = 0.672, and 95% CI = 0.485–0.930). In addition, the rs4938723 polymorphism was negatively associated with gastric cancer risk in the comparison model of C versus T (P_H_ = 0.843, OR = 0.758, and 95% CI = 0.643–0.893), CT versus TT (P_H_ = 0.381, OR = 0.755, and 95% CI = 0.598–0.953), CC versus TT (P_H_ = 0.400, OR = 0.584, and 95% CI = 0.405–0.842), CC/CT versus TT (P_H_ = 0.664, OR = 0.715, and 95% CI = 0.574–0.892), and CC versus CT/TT (P_H_ = 0.254, OR = 0.667, and 95% CI = 0.471-0.943). The rs4938723 polymorphism showed also reverse correlation with esophageal squamous cell cancer in the comparison model of CC versus TT (P_H_ = 0.345, OR = 0.787, and 95% CI = 0.638–0.972) and CC versus CT/TT (P_H_ = 0.164, OR = 0.774, and 95% CI = 0.633–0.947).

### Publication bias and sensitivity analysis

**Figure 3:**
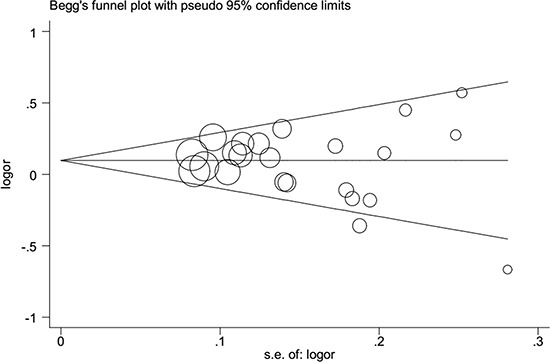
Publication bias tested by Begg's funnel plot in general population Models represented in overdominant model.

**Figure 4:**
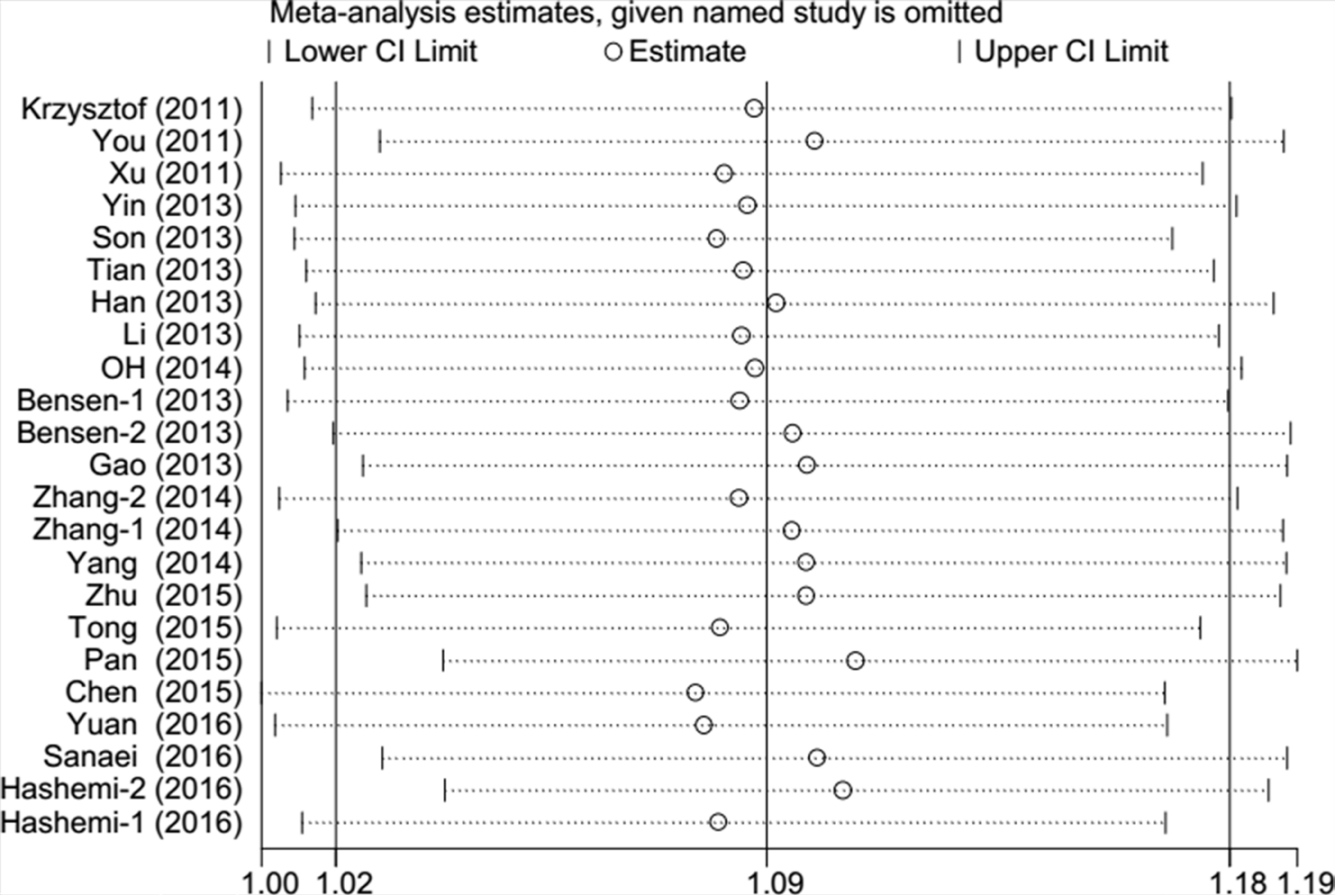
Sensitivity analysis of each study included in this meta-analysis by omitting each data set in the meta-analysis (overdominant model)

Begger's funnel plot and Egger's test were employed to evaluate the possible publication bias in our study. As shown in Table 2 and Figure 3, no evidence of publication bias was detected in all comparisons. Sensitivity analysis was performed to examine the influence of the individual data set to the pooled ORs. As shown in Figure 4, the pooled ORs and 95% CIs were not significantly altered when any part of the study was individually omitted. These data indicate that the results of our meta-analysis are reliable.

### Cumulative meta-analysis

**Figure 5:**
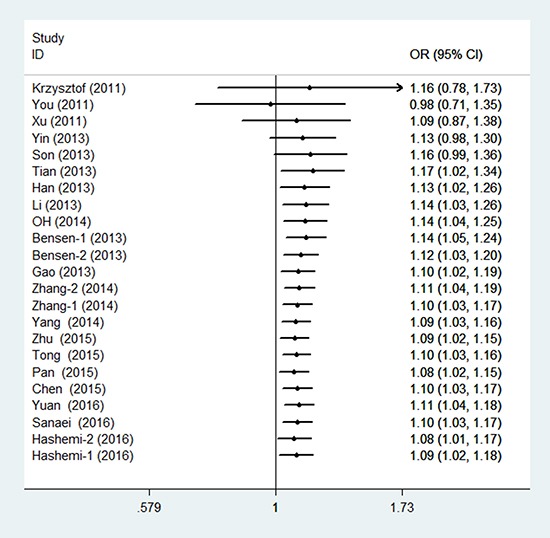
Cumulative analysis according to the chronological order (overdominant model)

According to the chronological order of the cumulative analysis, OR point estimates and confidence intervals were stable and exhibited a good change of trend, as shown in Figure 5.

## DISCUSSION

The miR-34 family is largely considered a tumor suppressor miRNA [34, 35]. miR-34b/c is located on chromosome 11q23, and has tissue-specific functions and different expression patterns in various cancers [36]. The miR-34b/c gene rs4938723 polymorphism has been investigated because of its potential association with the increased risk for cancer development [13–15, 17]; however, the results remain inconclusive. This updated meta-analysis was performed to obtain conclusive results about the association of rs4938723 polymorphism and cancer risk. A total of 10,812 cancer cases and 11,719 healthy controls were retrieved for the analysis. An increased risk for cancer was observed for the rs4938723 polymorphism under overdominant (CT vs. CC/TT) model. A stratified analysis showed that this association was observed in people with hepatocellular carcinoma, colorectal cancer, gastric cancer, and esophageal squamous cell cancer. Our results indicate that the rs4938723 polymorphism is a risk factor for cancer.

Several meta-analyses investigated the association between miR-34b/c gene rs4938723 polymorphism and cancer risk [11, 12, 37]. In the study conducted by Qiu et al., the meta-analysis included 11 studies, and indicated that allele C and genotype CT might be risk factors for hepatocellular cancer, and protective factors for colorectal cancer [37]. The meta-analysis conducted by Li et al. included 13 studies, and indicated that the rs4938723 polymorphism was associated with an increased cancer susceptibility of the Asian population. However, the polymorphism reduced susceptibility to colorectal cancer and esophageal squamous cell cancer in Asians [11, 12].

Nine studies have been conducted from 2014 to 2016 [13–9, 32, 33]. These studies comprised 3,059 cases and 3,705 controls and were included in our current meta-analysis. Thus, our meta-analysis increased the number of relevant studies compared to previous meta-analyses. Our study had relatively large study number, thus we could conduct more subgroup analyses based on different ethnicities and types of cancer. The statistical power of our meta-analysis was significantly increased. Consistent with the previous meta-analyses, we observed an increased risk for cancer that was associated with the miR-34b/c gene rs4938723 polymorphism using the overdominant model.

Different cancer types might contribute differently to the overall result of our meta-analysis. In our analysis, 12 types of cancers were included. In the stratified analysis, the rs4938723 polymorphisms were associated with an increased risk of hepatocellular carcinoma, but with a decreased risk for colorectal, gastric, and esophageal squamous cell cancer.

Some limitations in this study should be mentioned: First, only studies in English and Chinese were included. Second, the number of included studies in some cancer types, such as gastric cancer, was relatively small. Nasopharyngeal carcinoma, osteosarcoma, renal cell cancer, cervical cancer, prostate cancer, and papillary thyroid carcinoma were each included only once, making stratification impossible. Therefore, the statistical power might be insufficient to assess the relationship in these cancers. Third, the studies included were mostly performed in the Asian population; two studies were in the Caucasian population, and one study was performed in the African population. Thus, large-scale studies including different cancer types and populations should be conducted in the future. Finally, some important confounding factors that contribute to cancer susceptibility, such as age, gender, and smoking, were not included in the stratified analysis and should be analyzed in future.

## MATERIALS AND METHODS

### Searching and screening eligible studies

In the present study, we conducted a comprehensive search of the PubMed, Embase, Web of Science, Wanfang, and CNKI databases to identify all potentially eligible studies on rs4938723 polymorphism and cancer risk. The last search was updated on December 22, 2016, by using the following search terms: (pre-mir-34b/c OR pri-miR-34b/c OR mir-34b/c OR microRNA-34b/c OR rs4938723), (gene OR polymorphism OR allele OR variation), and (cancer OR carcinoma OR tumor). We also manually searched the references of previous meta-analyses and reviews to identify other studies. All of the selected studies in our meta-analysis conformed to all of the following criteria: (1) case–control studies; (2) evaluation of rs4938723 polymorphism and cancer risk; (3) sufficient genotype frequency data for calculating the OR and 95% CI; and (4) genotype distribution of the control group that was consistent with the HWE. The major exclusion criteria were as follows: (1) duplicates of previous publications; (2) studies irrelevant to cancer or miRNA-SNPs; and (3) no available data used for the SNPs.

### Data extraction and quality assessment

Two authors (HL and BXM) independently reviewed and determined whether an individual study was eligible for inclusion. A third reviewer would participate if any discrepancy was encountered, and a final decision was made by the majority of the votes. The following data were extracted from each eligible study: (1) the surname of the first author, (2) year of publication, (3) country, (4) ethnicity, (5) cancer type, (6) number of controls and cases, (7) allele or genotype frequencies of cases and controls, and (8) HWE of the control subjects. The quality assessment for each eligible study was assessed according to a methodological quality assessment scale that was extracted and modified from previous studies [38]. Six items were assessed. The quality scores ranged from 0 to 10.

### Statistics

The strength of the association between rs4938723 polymorphism and cancer risk was assessed by ORs and corresponding 95% CIs under five different genetic models. The models were as follows: allele model (C vs. T), genotype model (CT vs. TT and CC vs. TT), dominant model (CC/CT vs. TT), recessive model (CC vs. CT/TT), and over-dominant model (CT vs. CC/TT). *p* < 0.05 was considered statistically significant. We conducted subgroup analysis according to ethnicity and cancer type. Heterogeneity among studies was examined with Cochran's *Q* test and the *I*^2^ statistic [39]. A random-effect model was used if the *p value* of the heterogeneity tests was no more than 0.1 (*p* ≤ 0.1); otherwise, the fixed-effect model was used. Analysis was performed by using the STATA 11.0 (Stata Corporation, College Station, TX, USA).

## CONCLUSIONS

In summary, our study shows that the miR-34b/c gene rs4938723 is a susceptible locus for cancer. The rs4938723 polymorphisms are associated with an increased risk for hepatocellular carcinoma, but decreased risk for colorectal, gastric, and esophageal squamous cell cancer.

## Abbreviations

miRNAs: microRNAs
SNPs: single nucleotide polymorphisms
HWE: Hardy–Weinberg equilibrium
NPC: nasopharyngeal carcinoma
HCC: hepatocellular carcinoma
OS: osteosarcoma
CRC: colorectal cancer
BC: breast cancer
ESCC: esophageal squamous cell cancer
LL: lymphocytic leukemia
RCC: renal cell cancer
CC: cervical cancer
GC: gastric cancer
PTC: papillary thyroid carcinoma
